# How and where does the brain predict the when: a Bayesian approach to modeling temporal expectation

**DOI:** 10.1186/1471-2202-12-S1-P53

**Published:** 2011-07-18

**Authors:** Gaurav Malhotra, Emmanuel Daucé

**Affiliations:** 1 L'Institut des Sciences du Mouvement, Université de la Mediterranée, 13288 Marseille, France

## 

How does the brain learn to predict when an event is going to occur? We know from studies that vary the foreperiod – the time between a warning and a response stimulus – that people can model the temporal variability of stimulus onset, i.e. react faster when a stimulus is statistically more likely [1]. We also know that reaction time (RT) decreases with the passage of time, showing that people dynamically update the temporal expectation of the stimulus [2]. Recent neurophysiological investigations and brain lesion studies have also revealed that areas in the dorsolateral prefrontal cortex, inferior parietal cortex and posterior cerebellum perform functionally distinct roles in the generation of temporal expectations [3]. In the current study, we use both behavioral and neurophysiological findings to develop a theoretical account of the computational processes that underlie the generation of temporal expectations.

**Figure 1:**
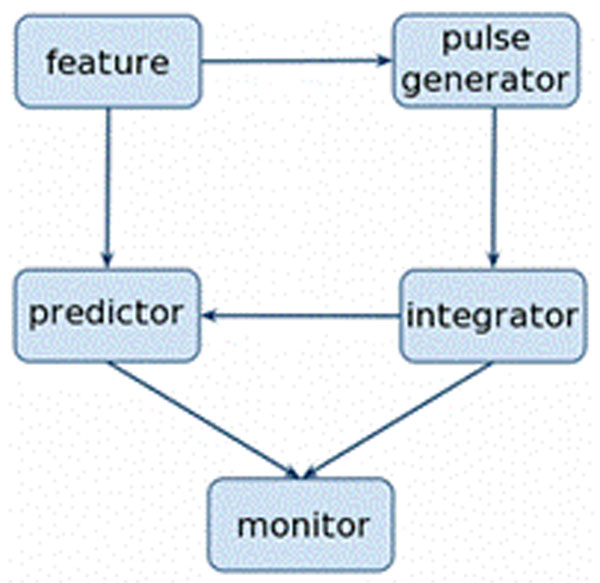
Independent stages in generation of temporal expectation.

We identify four independent processes that are critical to generating temporal expectation: (a) a pulse-generator (oscillator or other tonic activity) that relates the sensory stimulus to an internal signal (b) an integrator, that accumulates the tonic activity, generating a temporal percept, (c) a predictor, that forms a probabilistic model based on the combination of the sensory signal and its temporal percept, and (d) a process that monitors the temporal percept and dynamically updates predictions, generating a temporal expectation (see Figure 1). The novelty of our work lies in laying out the computational principles behind the processes (c) and (d). We propose that the brain learns to predict time by modeling sensory signals as a finite mixture of hidden temporal causes. The problem of prediction can then be translated to a Bayesian inference problem and learning can be performed through well known algorithms such as expectation maximization. The monitor updates predictions dynamically by moving through the sequence of temporal causes, a process that can be modeled as a Hidden Markov Model that progressively invalidates temporal causes with the passage of time.

Simulating this model reproduces the faster RTs with increasing foreperiods for a rectangular distribution of foreperiods and the lack of change in RTs when using an exponential distribution of foreperiods, a well known behavioral finding [2]. In addition to these implicit timing results, simulations on the model also reproduce explicit timing results where top-down, cue-based learning is used to orient participants' attention in time [4]. Thus, our research identifies the distinct computational steps involved in the generation of temporal expectation. Prediction itself seems to require a forward modelling of sensory signals, a function typically attributed to the parietal cortex and the cerebellum [5], while dynamically updating these predictions seems to require maintaining and moving through a sequence of hypotheses, a high-level cognitive function typically attributed to the prefrontal cortex. Thus, by laying out the computations underlying each process, our study paves the way for understanding the role of different regions of the brain in predicting the time at which to expect a stimulus.
